# The value of 3D reconstructions in determining post-operative reduction in acetabular fractures: a pilot study

**DOI:** 10.1007/s00068-019-01148-8

**Published:** 2019-06-01

**Authors:** Elke Arts, Han Nijsink, Luc Verhamme, Jan Biert, Mike Bemelman, Lars Brouwers, Bas van Wageningen

**Affiliations:** 1 Department of Trauma Surgery, Radboud University Medical Centre, Nijmegen, The Netherlands; 2 3D Lab, Department of Maxillofacial Surgery, Radboud University Medical Centre, Nijmegen, The Netherlands; 3 Department of Trauma Surgery, Elisabeth-Tweesteden Hospital, Tilburg, The Netherlands

**Keywords:** 3D reconstruction, Fracture reduction, Acetabular fractures, 3D modelling

## Abstract

**Background:**

In patients with acetabular fractures, the reconstructed three-dimensional (3D) model of the contralateral acetabulum could be used as a mirrored template for the anatomical configuration of the affected joint. This has not been validated.

**Objective:**

To investigate whether the right and left acetabula, as reconstructed 3D models, are valid mirrored duplicates that can be used as a reference model for the contralateral side.

**Methods:**

CT scans of twenty patients with unaffected acetabula were used. The symmetry of the generated 3D models was evaluated through: (1) mirroring of the acetabulum; (2) initial rough matching; (3) automatic optimisation of the matching via surface-based matching; (4) calculation of distances between surfaces by evaluating the Euclidean (straight-line) error distance between the closest points between left and right. The percentages of surface points of the left and right acetabulum with a distance smaller than 0.5, 1.0, 1.5 and 2.0 mm were calculated and evaluated, in relation to Matta’s criteria, for acetabular fracture reductions.

**Results:**

The mean distance deviation was less than 0.75 mm in all 40 comparisons. The calculated distances in 90.7% of the surface points of the left and right acetabulum were below the tolerance threshold of 1.0 mm, based on Matta’s anatomical reduction criteria, and 98.7% of the surface points scored below Matta’s imperfect tolerance threshold of 2.0 mm.

**Conclusion:**

This study demonstrates 3D reconstructed models of healthy left and right acetabula are highly similar and could potentially be used as mirrored duplicates. The next step will be to investigate these results in patients with reduced acetabular fractures.

## Introduction

Acetabular fractures are frequently the result of high-energy injuries [1, 2]. In an ageing but active population, the incidence of acetabular fractures in elderly patients due to minor trauma is increasing [3–6]. Most acetabular fractures are classified using the Judet and Letournel classification system [7]. In most cases, treatment of acetabular fractures requires an open surgical fixation of displaced fragments, for which several different approaches and techniques have been described [7–11]. A common long-term complication is osteoarthritis, which occurs in up to 20% of patients with displaced acetabular fractures. An important factor influencing the risk of hip osteoarthritis and functional outcome is post-operative joint congruity. This is of particular importance in fractures that involve the weight-bearing dome of the acetabulum [1, 2, 12]. A post-operative assessment of fracture reductions is performed using objective radiographic markers of displacement. The criteria by Matta et al. [13], which focus on any residual displacement of columns, walls and the superior dome, are often used for this purpose [12, 13]. According to these criteria, a post-operative reduction is graded as anatomical (≤ 1 mm of displacement), imperfect (2 to 3 mm of displacement) or poor (> 3 mm displacement). These objective measures of anatomical reduction and stable fixation of acetabular fractures serve as predictors for joint functional outcome, particularly in the long term [13–16]. Originally, post-operative assessments of fracture reduction were performed using plain radiographs. However, computed tomography (CT) scans provide more accurate information on intra-articular (bone) fragments, residual articular steps and gaps at the joint surface, as well as the severity of marginal impaction. Furthermore, the acetabulum can be viewed from different angles, which provides additional details on the accuracy of the fracture reduction [17, 18]. Despite these advantages, the process of understanding fracture patterns and assessments of fracture reductions on two-dimensional (2D) CT-generated images remains difficult. The objectivity and reliability of fracture assessments and classifications using 2D CT images have been questioned, and certain radiographic markers, such as the teardrop landmark, are challenging to identify accurately [19–21]. Consequently, it is difficult to form evidence-based recommendations based on these radiographic markers, as the (long-term) outcomes of the objectified residual articular incongruity have not been clearly demonstrated [21]. A recent study described limited inter-observer reliability for CT scans in the assessment of post-operative acetabular fractures. A complicating factor herein is the lack of a specific standardised measurement technique for assessing post-operative acetabular fractures using CT scans [18, 21]. The addition of three-dimensional (3D) reconstructions has gained popularity in the identification of fracture patterns and as a training tool [22]. It was found that 3D CT reconstructions are easier to interpret than axial CT images [22]. Furthermore, reconstructed 3D models, once they are validated, could also be used as a less complicated, standardised and more reliable method of post-operative acetabular fracture reduction and joint congruity assessment. Thus, it is hypothesised that the contralateral side could be used as a mirrored template for the anatomical configuration of the affected joint. If this method proves to be valid, it could be used as an objective fracture reduction assessment tool. This technique was reported to produce acceptable accuracy and repeatability for other modalities, but not for 3D models [23, 24]. Therefore, the objective of this study is to investigate whether the right and left acetabula, as reconstructed 3D models, are mirrored duplicates that can be used as a reference model for the contralateral side.

## Methods

### Data acquisition and generation of acetabular 3D reconstructions

Twenty CT scans of patients with pelvic ring fractures, having unaffected acetabula, were used in this study. The scans were acquired by a *Siemens Somatom 64* scanner (Siemens Healthcare GmbH, Erlangen, Germany). A reconstruction protocol, with a slice thickness of 0.5 mm and soft reconstruction filters, was used to generate high-resolution image data and to minimise soft tissue image noise. This study was exempted from the scope of the Medical Research Involving Human Subjects Act (WMO), according to our institutional ethics committee.

The data were saved in the Digital Imaging and Communication (DICOM) format and extracted anonymously from the picture archiving and communication system (PACS). The *Philips Intellispace Portal* (Philips, Amsterdam, Netherlands) was used to render the DICOM data into 3D reconstructions and to remove the femoral head. Furthermore, the 3D model was cleaned of small objects, introduced by artefacts or soft tissue remnants, in the *Philips Intellispace Portal*. The models were saved as STL (surface tessellation language) files. Using *MeshLab* [25], left and right acetabular surfaces were extracted from the pelvic structure, in order to minimise computation time in the following steps.

#### Mirroring and registration

For evaluating the symmetry between surfaces of two contralateral anatomical structures, four steps were necessary: (1) mirroring of one structure; (2) initial rough matching; (3) automatic optimisation of the initial matching; and (4) calculation of the distances between surfaces of both models.

**Fig. 1:**
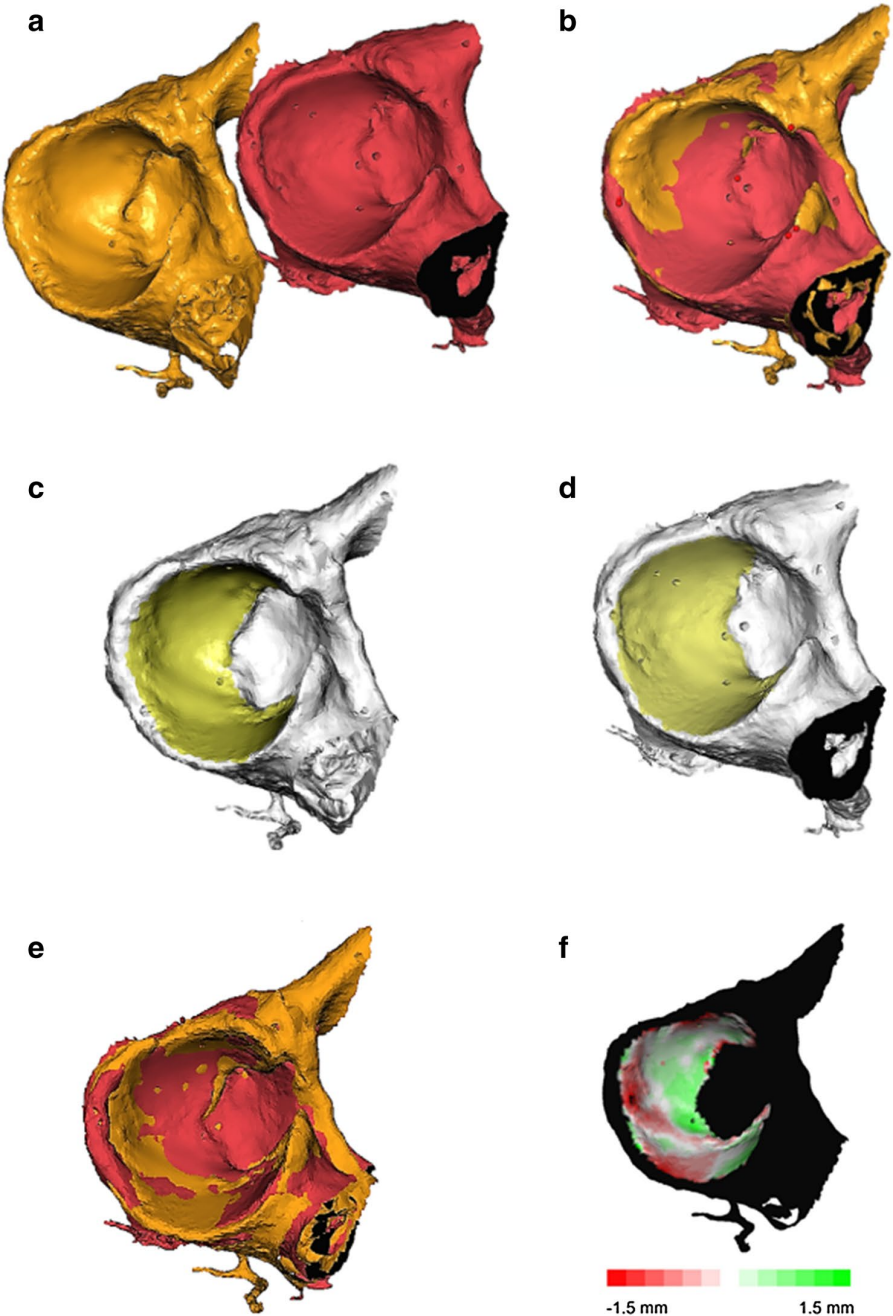
Surface registration and creation of a distance map in patient 12. **a** Situation before registration. The red surface represents the original unmirrored right acetabulum, whereas the orange surface is the mirrored contralateral left acetabulum (LM2RO registration). **b** Initial rough registration using point-based registration. **c**, **d** Selection of the articular surface of the acetabulum (orange) used for the ICP algorithm in both 3D models. **e** Refined matching as a result of applying the surface-based registration. **f** Distance map displaying the distances between the closest points of the articular surface in both models. Black parts in the model are excluded from further analysis, as they are not part of the articular surface

(1) Using *Maxilim v2.3.0.3* (Medicim NV, Mechelen, Belgium), one side was mirrored to facilitate comparison with the contralateral acetabulum (Fig. 1a). (2) A point-based matching algorithm was used to preregister this mirrored acetabulum with the unmirrored counterpart (Fig. 1b). (3) After this initial registration, a surface-based matching of the articular surface of the acetabulum was performed to optimise the matching (Fig. 1c–e). The articular surface was defined by the sharp edge of the acetabular labrum and the delineation of the acetabular fossa. This used registration method was an adapted version of the iterative closest point (ICP) algorithm and aimed to iteratively minimise the distance between points in two surface models [26]. (4) After the surfaces were aligned, the symmetry between both acetabula was expressed by evaluating the Euclidean (straight-line) distance error between the closest points in both models. Therefore, the articular surface of the mirrored model was selected and the Euclidean distances to the closest points in the unmirrored surface were calculated and displayed using a distance map (Fig. 1f).

In order to evaluate intra-observer agreement of the registration method, both the registration steps and the selection of the region of interest for the distance map were performed twice in all patients. For each patient, the mirrored left acetabulum was first matched onto the right original structure, which we designated as the left mirrored to right original (LM2RO) registration. Second, the right mirrored to left original (RM2LO) registration was obtained by matching the mirrored right acetabulum onto the left unadjusted (original) acetabulum. The difference between both procedures was used to evaluate the error introduced by the manual steps in the registration process. To determine the inter-observer agreement, the procedures were repeated by a second assessor for the first ten patients. Results were then compared between the first and second assessor. Both of the assessors followed the same instruction, and none of the assessors were specifically trained prior to performing this task.

### Data analysis

The Euclidean error distances from the distance map were exported and used to calculate symmetry parameters, such as the mean of the error distances, standard deviation (SD) and the 95th percentile, using *MATLAB* (Mathworks, Natick, MA, USA). Box-and-whisker plots were generated to summarise the calculated distances for each patient. In the analysis, the absolute values of the Euclidean distances were used.

#### Registration variability

Descriptive statistics were used to test whether the difference between the mean distances for the two registration methods (LM2RO and RM2LO) was significant. A Kolmogorov–Smirnov test was used to test for normality. In the case of a normal distribution, the unpaired *t* test was used. The Mann–Whitney *U* test was used when the data distribution was not normal. A *p* value of 0.05 was determined as significant. To determine inter-observer agreement, the mean distances (mm) between the mirrored and the original models, for both registration methods (LM2RO and RM2LO), were compared between assessor 1 and 2. The mean distances were also categorised according to Matta’s criteria for acetabular fracture reductions (< 0.5, 0.5–1, 1–1.5 and > 2 mm). The percentage of agreement between assessor 1 and 2 was calculated using these categorised data.

#### Symmetry representation

To generate a comprehensive symmetry value, the percentage of points from the surface of interest having a distance smaller than a certain tolerance threshold was calculated. The selected thresholds were 0.5, 1.0, 1.5 and 2.0 mm, based on the aforementioned Matta’s criteria for acetabular fracture reductions, considering deviations smaller than 1.0 and 2.0 mm being anatomical or good reductions, respectively [13].

## Results

### Patient characteristics

Patients had a mean ± SD age of 39.6 ± 15.6 years of age. A total of 14 (56%) included patients were male, and 80% of all cases were multi-trauma patients. The acetabulum was non-injured on both sides in all patients. None of the included patients had a recorded history of acetabular fractures or joint disease.

### Registration deviation

**Fig. 2:**
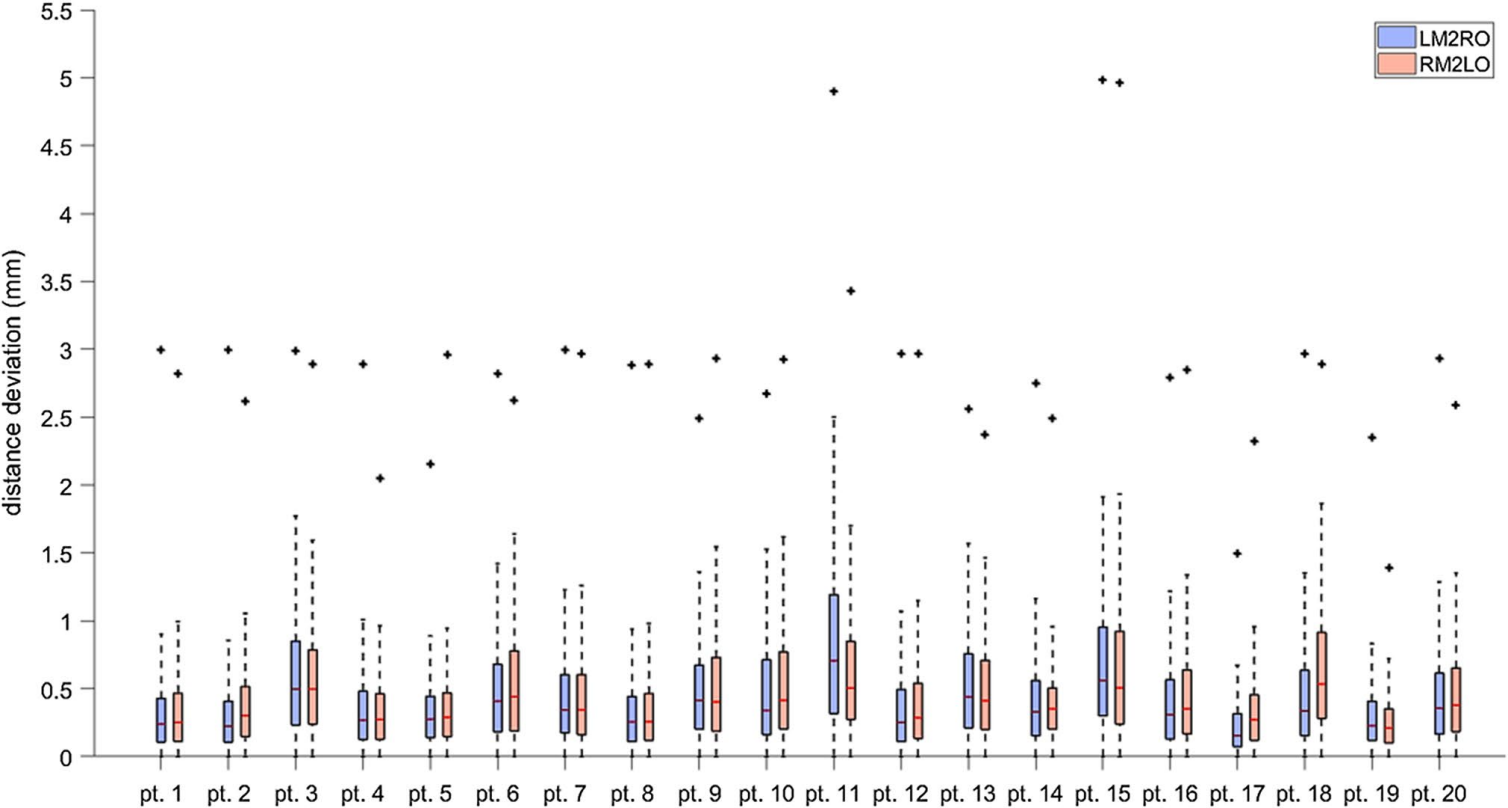
Boxplot of the calculated distances between the two 3D models. Blue represents LM2RO registration, and red represents the RM2LO registration. The asterisk symbols represent the maximal values in the data set

Box-and-whisker plots of the Euclidean error distances for the 20 patients are summarised in Fig. 2. The blue plots show the data for the situation when the left mirrored acetabulum was matched on the original contralateral acetabulum (LM2RO), whereas for the red plots, the registration order was switched (RM2LO).

The mean distance between the two models was below 0.75 mm for all patients. The mean Euclidean error distance for both registration methods combined was 0.46 ± 0.43 mm. The mean distance deviation was smaller than 0.75 mm in all 40 comparisons. A summary of the data is reported in Table 1.

**Table 1:** Mean error, standard deviation, 95th percentile and the average of the maximal deviations for the symmetry comparison computed for both separate registration groups and the combination of groups

Registration group	Mean error (mm)	Standard deviation (mm)	95th percentile (mm)	Average of maximal deviations (mm)
RM2LO	0.45	0.44	1.28	2.93
LM2RO	0.46	0.42	1.24	2.80
LM2RO and RM2LO	0.46	0.43	1.26	2.86

### Registration variability

**Fig. 3:**
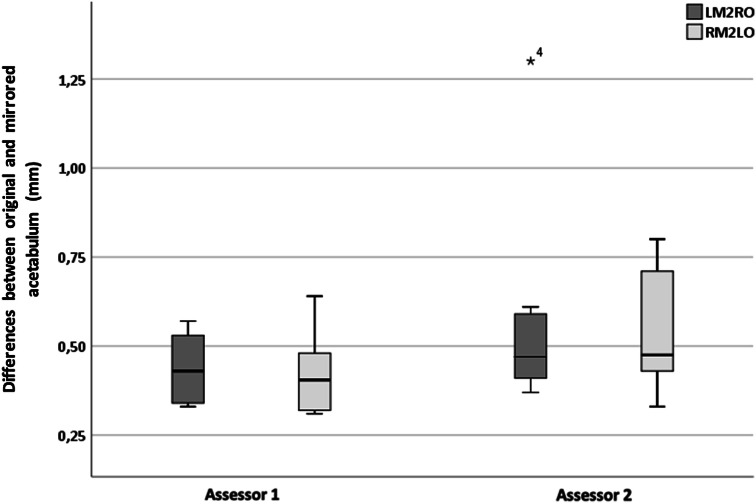
Boxplot of the calculated distances between the two 3D models (LM2RO and RM2LO) for assessor 1 and assessor 2

The mean distance measurements for the twenty patients were not normally distributed, proven by the Kolmogorov–Smirnov test (*p* < 0.001). A Mann–Whitney *U* test showed that the mean distance between mirrored and original models calculated for both registration methods was not significantly different (*p* = 0.36). For the inter-observer agreement analyses, nonparametric testing was used to compare the mean distance measurements generated by the manual input of assessor 1 and 2. The overall median(p25–p75) distance for LM2RO registrations was 0.43 mm (0.34–0.53) for assessor 1 and 0.47 mm (0.41–0.60) for assessor 2, which was not significantly different (*p* = 0.14). For RM2LO, the median difference was 0.41 mm (0.32–0.49) for assessor 1 and 0.48 mm (0.43–0.71) for assessor 2, which was not significantly different (*p* = 0.09). All measured distances between the mirrored and original models were < 1 mm, with the exception of 1 value (patient 2 LM2RO; 1.30 ± 1.03), and the absolute difference between assessors averaged well under 0.5 mm (Fig. 2). Overall, percentage of agreement was moderate at 70%1 (Fig. 3).

### Symmetry representation

**Fig. 4:**
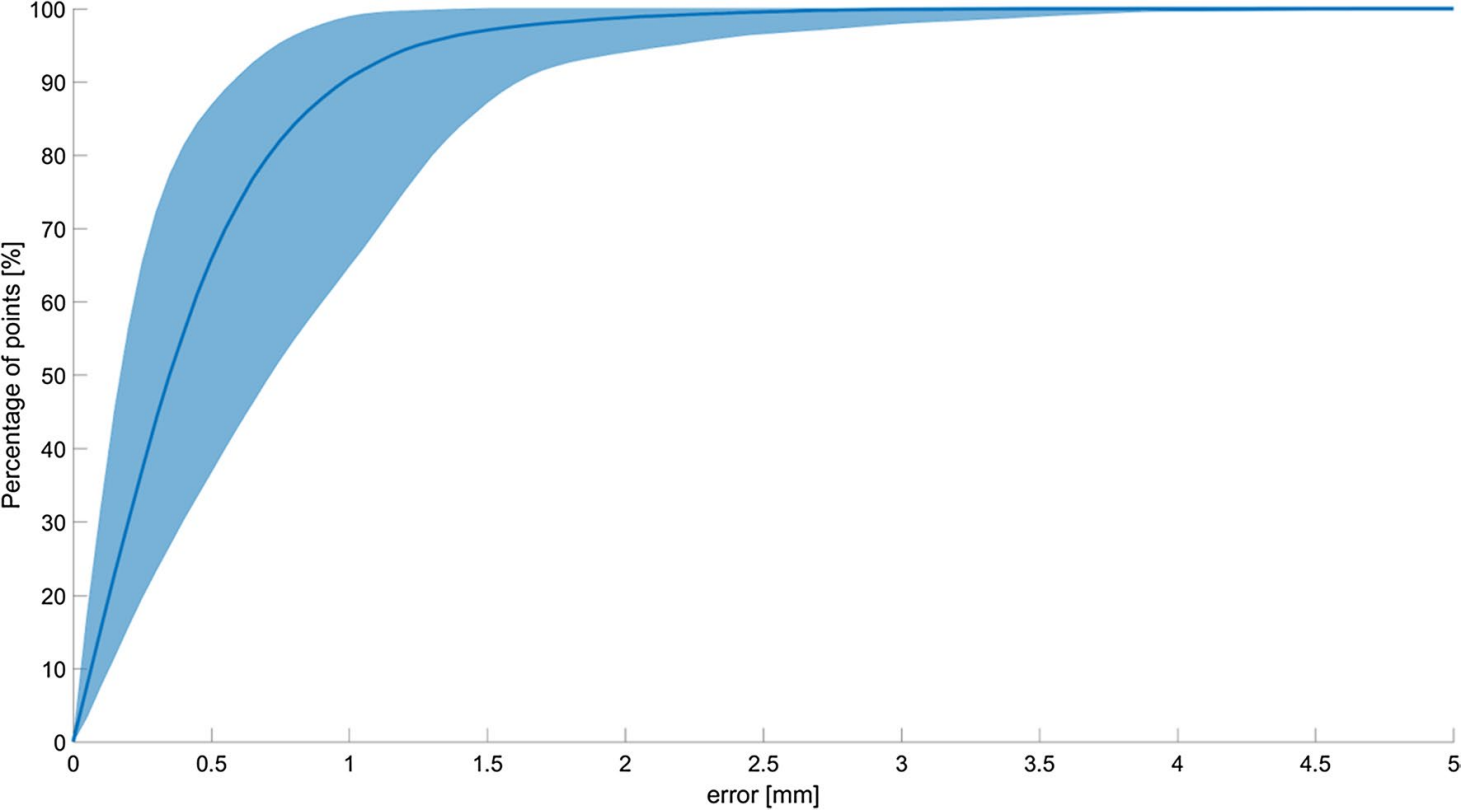
A cumulative distribution plot illustrating the percentage of points below a distance deviation. The blue line represents the average error for all 40 registrations, and the blue area represents the variance within all 40 symmetry comparisons

A cumulative distribution plot was generated to visualise the relation between the distance error and the surface percentage (Fig. 4). The figure displays the cumulative percentage of surface points for each distance deviation.

A summary of the data is reported in Table 2. It can be deduced that, on average, more than 90% of the surface was scored as being anatomical using Matta’s criteria. A total of 98.7% of the surface was scored below Matta’s imperfect tolerance threshold of 2 mm.

**Table 2:** Symmetry parameters calculated for both groups (LM2RO and RM2LO)

Tolerance threshold (mm)	Mean surface (%)	Standard deviation (%)	Maximal surface (%)	Minimal surface (%)
0.5	66.7	12.8	86.7	37.5
1	90.7	7.3	99.0	64.8
1.5	96.9	3.1	100	87.4
2	98.7	1.6	100	94.1

**Fig. 5:**
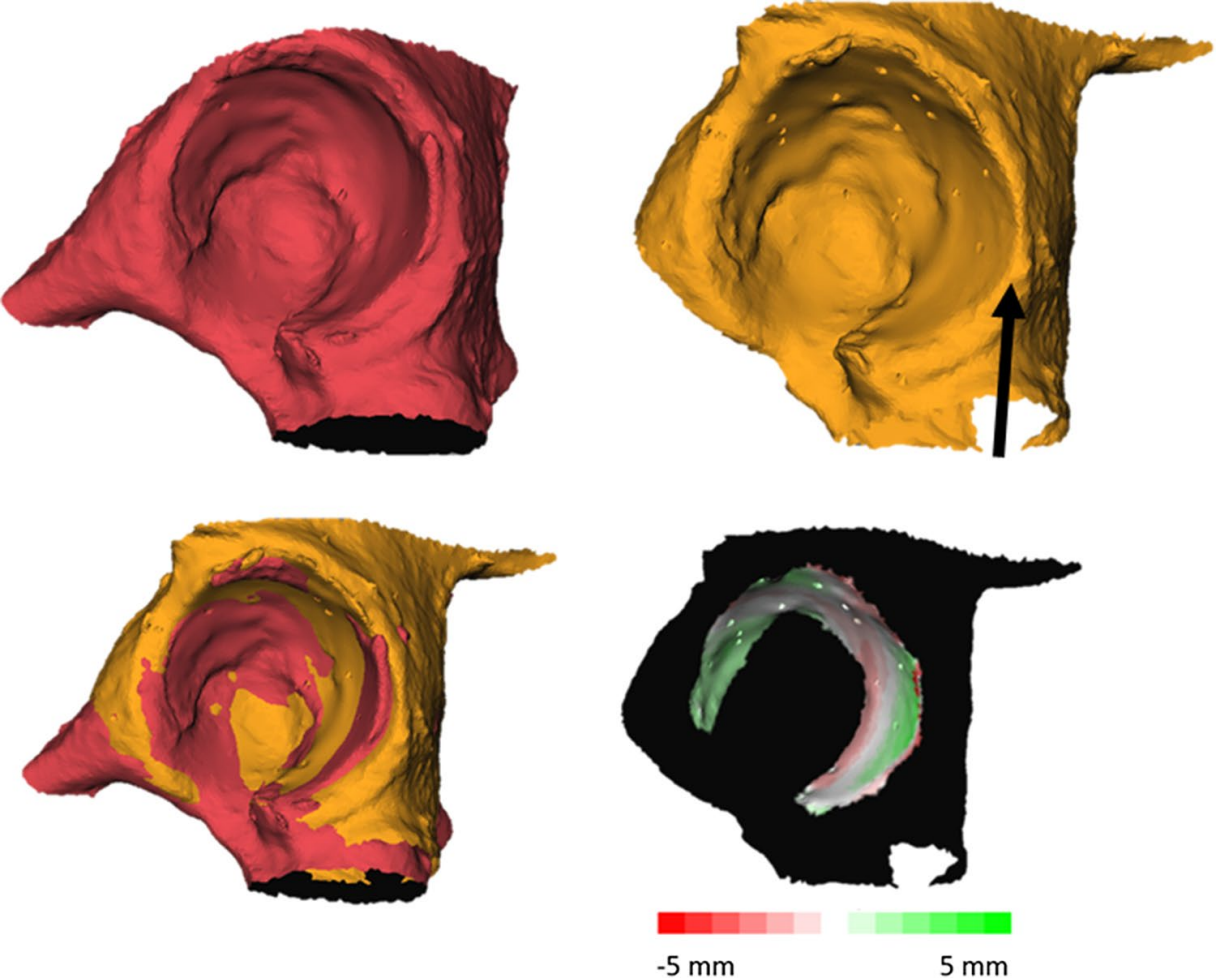
Low symmetry seen in patient 11 (RM2LO registration). The image possibly shows erosion (black arrow) at the lateral side of the left acetabular cup (orange model)

Out of all 20 patients, the symmetry was lowest for patient 11. A mean deviation of 0.73 mm was seen. Figure 4 shows the registration between the left and right acetabulum for this case. The results are influenced by the differences seen at the lateral side of the acetabular cups. The left acetabular cup is dilated, which resulted in the large deviation calculated for this patient (Fig. 5).

## Discussion

The results of this study showed great similarity between 3D reconstructed models of right and left healthy acetabula. After registration of both 3D models, the mean distance deviations between both models were below 0.75 mm. Furthermore, the mean surface percentage with a distance deviation below Matta’s imperfect criteria of 2 mm is 98.7%. The method proved to be robust, since two consequent registration procedures did not significantly influence the results, with adequate to good inter-observer agreement.

The results showed a high degree of symmetry between the left and right acetabula, with 90.7% of surface points deviating less than 1 mm between both models. When considering Matta’s criteria, this would translate to a deviation that is below the tolerance threshold of 1 mm, based on Matta’s anatomical reduction [13]. Hence, these results indicated that the left acetabulum is a representative model for the right acetabulum, and vice versa. In addition, the symmetry analysis appears to be able to detect small differences between both acetabula, as demonstrated in this study. This has been clearly demonstrated in patient 11, since a minor difference in the lateral wall between the acetabula showed a notable increase in the mean Euclidean distance deviation (Fig. 4). The use of 3D models of the acetabulum in the preoperative setting is steadily gaining more interest. It may also have potential as a technique in post-operative fracture reduction assessments [24–26]. Currently, 2D CT-generated images are frequently used for this purpose; however, there is no widely accepted standardised method describing exactly how to measure fracture displacements using this modality [18, 21]. The lack of standardisation hinders reproducibility and validation, particularly in relation to functional outcomes [18–21]. Compared to 2D axial CT images, 3D CT images were found to be easier to interpret [22]. Furthermore, the results of this study indicated that the mirrored CT-generated 3D models of one side of the acetabulum can be used as a duplicate for the other side. In patients with acetabular fractures, the mirrored 3D model of the unharmed, healthy acetabulum could potentially be used as an anatomical model for the fractured side in post-operative fracture reduction assessments. Therefore, a more standardised method, such as the one proposed in this study, may provide a substantial advantage in the post-operative setting as a tool for fracture reduction assessments.

Several limitations of this study need to be considered. Certain pre-existing patient characteristics that affect the structure or integrity of the acetabulum could influence the symmetry analysis, particularly, if these characteristics are asymmetrical, such as arthrosis of the hip joint or a previous injury. The presence of osteosynthesis materials in the pelvic area could lead to imaging artefacts, which could influence the segmentation of the 3D models and therefore the results of the symmetry analysis. Also, characteristics of the used software could affect the results. Different software packages and segmentation techniques for the generation of 3D models are available. These different segmentation methods will influence the results of the symmetry analysis [4, 5]. Therefore, factors such as user-friendliness and segmentation quality should be evaluated before choosing a method. In this study, the segmentation was performed using Philips Intellispace Portal, since this package was found to be easy to use due to the direct link with PACS and the fast, automatic generation of the 3D models. This automatic generation reduced the manual influence in the segmentation process, thus improving the overall robustness of the method. On the other hand, manual input is required in the registration procedure, which influences the final match and the surface comparison while also possibly introducing inter-observer variability. Therefore, in this study, intra-observer agreement was analysed by repeating the symmetry analysis in all patients with a reversed order (RMLO and LMRO registration) which generated similar results. Furthermore, the analysis in the first ten patients was repeated by a second assessor who had no prior experience with the analysis and the software program. Mean distance measures generated by manual input from both assessors showed great similarity between the mirrored and original model, and these values did not differ significantly between assessor 1 and 2. Some inter-observer variation was present in these data; however, these observed differences were relatively small with individual mean distance measurements of both observers falling well within the threshold for anatomical reduction according to Matta’s criteria for acetabular fracture reduction (1 mm). Hence, these differences are unlikely to be clinically significant. Overall, these results indicate moderate inter-observer agreement, and residual variability in manual input for the registration procedure is unlikely to significantly influence the symmetry analysis.

The Maxilim software package was used for the mirroring and registration steps, since these functionalities are easy to use. With a set of clear instructions, it is likely that everyone would be able to perform the symmetry analysis within 30 min. Further automation of the total procedure will minimise inter- and intra-observer variability, will simplify and objectify the method and will increase overall reliability.

In conclusion, this study demonstrates that 3D reconstructed models of healthy left and right acetabula are highly similar, showing great potential for left and right acetabula to be used as mirrored duplicates. Further research is required to repeat these results. The next step will be to investigate this method in patients with unilateral acetabular fractures, as a tool for post-operative assessment of fracture reduction. Also, the value of the surface symmetry parameters used in this study as potential predictors of clinical outcomes requires further investigation.
